# A novel hybrid model for identifying the most informative instances for improving text data classification

**DOI:** 10.1371/journal.pone.0355603

**Published:** 2026-08-10

**Authors:** Amira Abdelwahab, Mohamed Salama

**Affiliations:** 1 Department of Information Systems, College of Computer Science and Information Technology, King Faisal University, Al-Ahsa, Saudi Arabia; 2 Department of Management Information Systems, Higher Institute for Specific Studies, Future Academy, Heliopolis, Cairo, Egypt; West Pomeranian University of Technology, POLAND

## Abstract

The rapid growth of user-generated textual content on the internet has intensified the need for accurate and scalable text classification methods. However, supervised learning approaches remain heavily constrained by the high cost and effort required for manual data annotation, particularly in large and heterogeneous datasets. To address this challenge, this paper proposes a novel hybrid active learning framework for efficient classification of unlabeled text data. The proposed approach integrates multiple classical machine learning classifiers—Support Vector Machines, Logistic Regression, Naive Bayes, and Random Forest—within a hybrid ensemble architecture, combined with a pool-based active learning strategy to iteratively select the most informative unlabeled instances for annotation. Textual data are transformed into numerical representations using several feature extraction techniques, including Bag-of-Words, TF-IDF, Word2Vec, and BERT-based embeddings, allowing for a comprehensive evaluation of representation effectiveness. Extensive experiments are conducted on four diverse benchmark datasets from healthcare, finance, spam detection, and e-commerce domains. The results consistently demonstrate that the proposed hybrid active learning model outperforms traditional ensemble classifiers across all datasets and evaluation metrics. In particular, TF-IDF-based hybrid ensembles achieve the highest gains in accuracy, precision, recall, and F1 score, while requiring substantially fewer labeled instances. Furthermore, the proposed framework exhibits strong robustness in imbalanced classification scenarios, significantly improving minority class detection. Overall, the findings confirm that combining hybrid ensemble learning with active learning offers an effective, lightweight, and cost-efficient alternative to purely transformer-based approaches, making it well-suited for real-world text classification tasks where labeled data are scarce or expensive.

## 1. Introduction

Active learning is a machine learning method to enhance the learning process’s efficiency by carefully picking the most informative samples for labeling. Through a repetitive process of selecting and annotating unlabeled data points, active learning algorithms can diminish the quantity of labeled data necessary for model training, all the while preserving or potentially boosting its performance [1]. With the widespread expansion of Internet services, there has been a substantial increase in the number of Internet users. This surge has led to a vast and diverse array of content accessible on the Internet. Consequently, the field of text data extraction and categorization has become a prominent area of study in today’s world.

A major issue in machine learning is that data can be very diverse and have many dimensions. However, using feature selection to reduce dimensions shows promise for overcoming this. Communication methods that depend heavily on text, like email, web pages, documents, and text messaging, are very important in this area. Supervised learning, where models are trained on labeled data, is the most common and successful tactic for tackling machine learning problems.

The utilization of supervised machine learning techniques, founded on pre-existing labeled or annotated datasets crafted by experts, enables the derivation of general rules applicable to a wide range of datasets. Over the past decade, algorithmic advancements have been nothing short of remarkable [2].

However, it’s crucial to recognize that the performance of models is highly contingent on data quality and quantity, which translates to significant investments in time, finances, and human resources. Consequently, challenges related to the analysis of Unlabeled data may arise.

The evaluation of Unlabeled data through machine learning approaches has become feasible through the enhancement of necessary preprocessing procedures tailored to the unique characteristics of textual data. Proper analysis necessitates preprocessing methods like language-specific tokenization, normalization, and stemming. Nevertheless, the supervised learning process demands the manual annotation of substantial volumes of data by human experts, making it a costly and time-consuming endeavor. Active learning emerges as a valuable approach, reducing the costs associated with human labeling while ensuring the accuracy and stability of supervised modeling [3].

Large amounts of varied data make manual classification infeasible because of the huge time, money, and resource costs. Machine learning methods for text classification have become very popular lately since they allow many kinds of data to be categorized automatically [4]. The main idea is that experts carefully pick and label some examples as a training set. Then a supervised learning model can use this labeled data to predict categories for the full dataset.

As a result of these efforts, active learning techniques have emerged as a valuable resource for achieving the required level of accuracy without the need to annotate or learn from an entire dataset. This provides a chance to improve the analysis of machine learning projects that use diverse data. These kinds of projects have Traditionally been very difficult because of the high costs, need for technical skills, and contextual knowledge required by human labelers.

**Fig 1:**
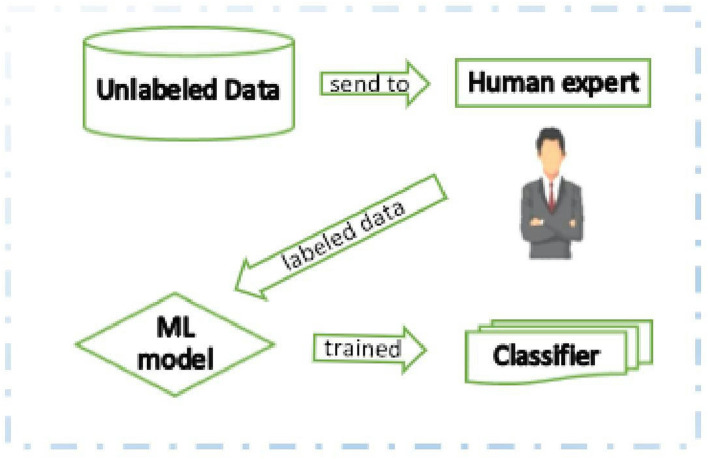
Traditional machine learning techniques.

**Fig 2:**
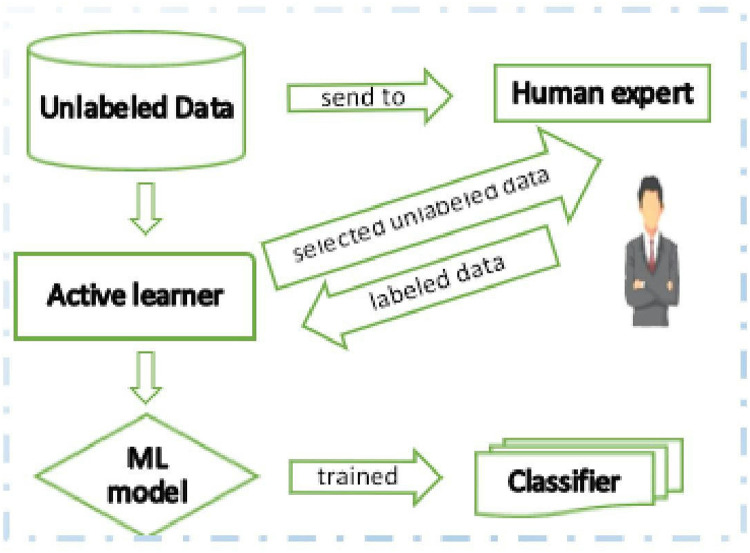
Active Machine learning techniques.

Traditional machine learning algorithms often necessitate a significant volume of pre-labeled data, as depicted in Fig 1. In contrast, active learning follows a distinct strategy. Rather than processing the entire dataset in one go, active learning entails soliciting a human expert to label only a small portion of the data points. An algorithm identifies the most informative instances using specific criteria and sends them to a human expert for labeling, as demonstrated in Fig 2. The expert then supplies the labels for these selected samples

Our goal is to develop an approach for efficiently managing the process of labeling text data for classification. Rather than randomly selecting text instances to label, which requires lots of labeled data to reach a desired level of accuracy, we want to strategically choose a small set of informative examples to label. This is the core idea behind active learning for text classification. Table 1 shows the difference between standard machine learning, which uses randomly sampled labeled data, versus active learning, which tries to minimize the labeling effort by selecting the most useful examples to label.

**Table 1:** Traditional machine learning versus active machine learning.

	Traditional Machine Learning	Active Machine Learning
**Sample of Data**	Randomly sample data from the pool of unlabeled data for training and testing purposes.	Choose data for a training set using a specific query strategy.
**Labeling process**	Label the chosen data with the assistance of a human expert.	
**Training process**	Train the machine-learning model	
**Evaluation process**	Check the model performance	

The key difference is that Traditional machine learning passively trains on randomly gathered labels, while active learning actively selects the data points that will be most valuable to the model if labeled [5]. This selective sampling enables active learning to produce high accuracy with lower data annotation requirements.

Active learning models achieve high accuracy with less labeled data. By actively targeting the labeling of critical examples, the dependency on large labeled training sets is reduced [6].

Text data needs to be converted into numerical representations before it can be used by hybrid classification models. Feature extraction is the process of transforming raw text into meaningful numerical features. However, this can negatively impact categorization performance if not done properly. In the context of textual data, typical feature extraction techniques encompass Bag of Words (BOW), Term Frequency-Inverse Document Frequency (TF-IDF), and Word2vec [7].

This research calls for developing an active learning framework that strategically leverages unlabeled instances to improve classification accuracy and efficiency. Challenges remain in applying active learning to unlabeled data, including intelligently sampling representative and informative examples from the unlabeled pool, accounting for skewed class distributions, handling noisy or unclear data points, and tailoring the active query approach to different types of classifiers. The central problem is devising an active learning technique that overcomes these hurdles to successfully exploit unlabeled data and reduce reliance on text.

To address these challenges, this paper proposes a novel hybrid active learning framework for text classification that combines ensemble learning with uncertainty-based sample selection. Instead of relying on a single classifier, the proposed approach integrates multiple heterogeneous learning algorithms—Support Vector Machines, Logistic Regression, Naive Bayes, and Random Forest—within a unified ensemble architecture. This hybrid design exploits the complementary strengths of different classifiers, leading to improved robustness and generalization under constrained labeling budgets. Furthermore, the framework systematically evaluates multiple feature extraction techniques within an active learning setting and demonstrates that TF-IDF consistently provides superior and more stable performance across diverse datasets.

In addition to improving overall classification accuracy, the proposed framework effectively addresses class imbalance by prioritizing uncertain and informative instances, particularly from minority classes, during the active learning process. This strategy enhances recall and balanced F1 scores without requiring additional resampling or cost-sensitive learning techniques. Extensive experiments conducted on multiple real-world datasets from healthcare, financial sentiment analysis, spam detection, and e-commerce domains validate the effectiveness, efficiency, and generalizability of the proposed approach. The results further show that the proposed hybrid active learning framework offers a computationally efficient alternative to transformer-based models, achieving competitive or superior performance under limited labeling budgets with significantly lower computational and annotation costs.

## 2. Related work

The classification of unlabeled text data poses a significant challenge due to the high cost and effort required to manually label large volumes of data. Traditional supervised learning approaches depend heavily on the availability of labeled datasets, which often limits their scalability and practical applicability. Consequently, reducing labeling requirements has become a critical research objective. Although unsupervised techniques such as clustering have been explored as alternatives, unlabeled data analysis remains an active research area because of its practical importance. Numerous studies have proposed methods to enable effective classification with fewer labeled instances by focusing on selecting only the most informative samples, thereby optimizing the tradeoff between annotation effort and model accuracy for heterogeneous data.

Random selection of unlabeled instances for manual annotation, commonly referred to as passive learning, often leads to suboptimal classification performance. Models trained using randomly labeled data typically suffer from lower accuracy due to inefficient sampling of the unlabeled pool. This limitation has motivated the adoption of more intelligent sampling strategies. Active learning addresses this issue by allowing the learning algorithm to iteratively select the most informative instances for labeling, leading to improved performance with significantly fewer labeled examples compared to passive learning approaches [8,9].

Various machine learning methods have been widely investigated for automated text classification, including k-Nearest Neighbors (k-NN), Naïve Bayes (NB), Decision Trees, Logistic Regression, and Support Vector Machines (SVMs) [10,11]. Despite their effectiveness, these methods face challenges related to high-dimensional feature spaces, sparsity, unstructured text, missing data, and model selection. Feature extraction techniques therefore play a vital role in improving classification performance. Commonly used representations include Bag-of-Words (BoW), Term Frequency–Inverse Document Frequency (TF-IDF), and Word2Vec embeddings. BoW represents text using word frequency vectors [12], and hybrid models based on BoW have demonstrated promising results [9]. TF-IDF refines this representation by emphasizing discriminative terms, and numerous studies confirm that TF-IDF often outperforms other feature extraction techniques [10,11]. In particular, SVMs and Logistic Regression combined with TF-IDF have achieved high classification accuracy [13], making TF-IDF a widely adopted choice for hybrid text classification frameworks [14,15].

Statistical classifiers such as NB, k-NN, and SVMs have been shown to be among the most successful techniques for text classification tasks [16,17]. Their performance can be further enhanced when combined with active learning strategies rather than passive random sampling. Additionally, for imbalanced datasets, Word2Vec embeddings integrated within hybrid models have demonstrated improved performance [18], while neural network–based hybrid approaches utilizing Word2Vec have also shown strong results [19].

Hybrid models, which combine multiple classifiers, have gained considerable attention due to their ability to leverage the strengths of different learning algorithms and improve generalization performance [20,21]. Recent research trends further emphasize the effectiveness of hybrid and attention-based architectures. For example, attention-driven multi-branch hybrid designs have been shown to enhance robustness and efficiency by adaptively fusing information from multiple components. Although primarily explored in computer vision, such principles provide useful insights for text classification and reinforce the motivation for adopting hybrid classifier ensembles.

In parallel, modern studies on active learning provide comprehensive guidance for designing effective query strategies. Active learning techniques focus on uncertainty sampling, diversity-based selection, and hybrid strategies that combine multiple criteria to identify the most informative samples [22,23]. These approaches have proven particularly effective for text classification, where labeling costs are high and data distributions are complex. Empirical evidence indicates that active learning can significantly reduce annotation effort while maintaining or even improving classification accuracy compared to passive learning [24,25].

Based on these observations, this paper employs an active learning framework integrated with a hybrid ensemble of classical classifiers and TF-IDF feature representation. The proposed approach aims to improve text classification performance when labeled training data is limited by selectively querying the most informative samples from a large pool of unlabeled data. By reducing dependence on large labeled datasets, the model enhances generalization capability while minimizing training cost and annotation effort.

## 3. Active learning approach and unlabeled data classification

Active learning has garnered much interest in reducing labeling costs in supervised learning by strategically choosing small, informative training sets [26]. It minimizes human effort by interactively querying the user to label only the most useful examples. By iteratively selecting small labeled datasets to train high-performance models [27]. This target labeling maximizes information gain. Active learning is thus well-suited when abundant unlabeled data is available.

The motivation behind active learning stems from the limitations and challenges associated with supervised learning. Labeling large amounts of data can be time-consuming, expensive, or even infeasible in some cases. Active learning provides a solution to these challenges by selectively choosing the most informative samples for annotation, reducing the reliance on labeled data, and improving the efficiency of the learning process.

### 3.1. Pool-based vs. stream-based active learning

Active learning can be categorized into two main approaches: pool-based active learning and stream-based active learning [28,29].

**Fig 3:**
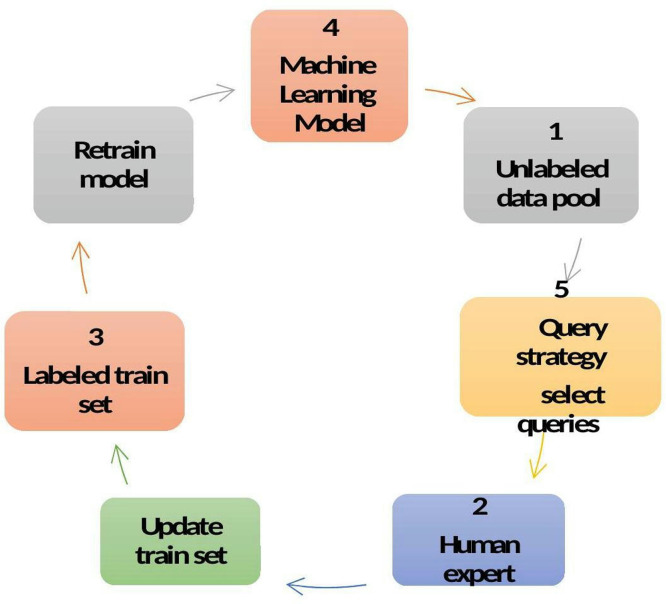
Active learning approach within a pool-based system for Unlabeled data.

**Fig 4:**
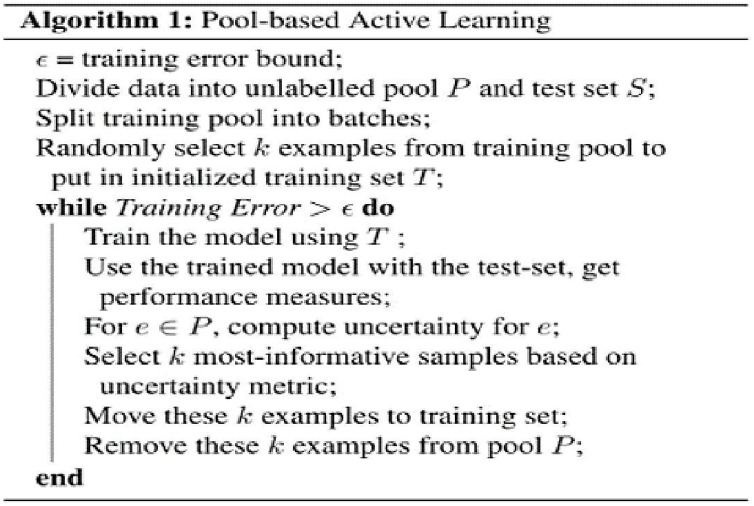
Illustrates the pool-based active learning process for unlabeled data.

The pool-based method is the most widely used active learning approach, where labeling and training occur in batches [30]. As shown in Fig 3, the learner is initially trained on a small labeled subset, then iteratively requests the most informative samples from the unlabeled pool to label and augment training. The general process is outlined in Fig 4.

In stream-based active learning, examples are provided sequentially [31]. Each instance must be either queried for a label or discarded immediately before viewing the next sample. There is no unlabeled pool, only a stream of examples. The algorithm interleaves labeling decisions on individual examples with training on the cumulatively labeled set.

The key difference is that pool-based learns in batch mode by sampling from a large unlabeled collection, while stream-based processes a sequence of examples individually in an online fashion. In both cases, the learner actively chooses which examples to request labels to maximize information gain and classification performance.

Active learning algorithms either batch sample useful instances from a pool to annotate (pool-based) or selectively query labels for streaming examples (stream-based) to minimize labeling costs while maximizing accuracy.

The quality of the data used significantly impacts classification performance. With abundant unlabeled data now available, the labeling process poses a major bottleneck. Moreover, data heterogeneity introduces new challenges in identifying the most useful examples to label. Active learning aims to address this by quantifying instance informativeness [32].

### 3.2. Query strategies in active learning

Query strategies are crucial in active learning by determining which instances to select for annotation. Various query strategies have been proposed in the literature, each with its advantages and limitations. Some commonly used query strategies [33,34] include:

*Uncertainty Sampling:* This strategy selects instances that the model is uncertain about. It can be further divided into different sub-strategies, such as least confidence, margin sampling, and entropy-based sampling. It selects the instances the model is least confident about predicting, measured by having probabilistic predictions close to 0.5 for the top two classes [35].

*Diversity Sampling:* This strategy aims to select diverse instances that cover different regions of the input space. It helps to explore the data distribution and reduce redundancy in the labeled dataset.

*Representative Sampling:* This strategy selects instances that are representative of the underlying data distribution. It aims to capture the different classes or clusters present in the data.

*Core-Set Sampling:* This strategy selects instances that maximize the model’s performance when labeled. It aims to select instances that are most informative for improving the model’s generalization.

The choice of query strategy depends on the specific task, dataset, and learning algorithm. Different strategies may perform better for certain scenarios, and a combination of strategies can be used to exploit their complementary strengths.

The goal of active learning is to build a model using the smallest possible labeled dataset to reduce expert involvement without hurting classification performance. These decreases labeling costs and time. The suggested hybrid active learning model is smart enough to choose which data to train on, but it’s still beneficial to train it on samples that significantly impact its performance

## 4. Methodology and experimental setup

This section provides an overview of the research methodology. It describes the proposed technique which involves dataset collection, preprocessing, feature extraction, hybrid classification, and evaluation procedures. Specifically, datasets are collected and preprocessed for analysis. Features are then extracted from the cleaned data to be used for modeling. hybrid classification methods which combine multiple models are utilized for prediction. Finally, evaluations are conducted to assess the performance of the proposed approach.

The research utilizes a pool-based active learning approach. Unlabeled data is divided into batches that are selectively sampled for labeling by a human expert. After each batch is labeled, the algorithm is retrained on the labeled data. This iterative process of sampling, labeling, and retraining continues until model accuracy reaches an acceptable level. For sample selection, an uncertainty sampling method is used to choose the most informative instances to label from the unlabeled data pool. After each round of labeling, model accuracy is evaluated to determine if additional labeling is needed. Once sufficient accuracy is achieved, the final labeled dataset is used to train a classifier.

### 4.1. Dataset description

We have gathered data from four different domains, which consist of datasets of different sizes and sources. These datasets are abbreviated as follows: HCR dataset (Health Care Reform), FPB dataset (Financial Phrase Bank), SSC dataset (SMS Spam Collection), and TBS dataset (Textbook Sales). In this subsection, we will provide a summary of each of these datasets.

The first dataset, known as the Health Care Reform dataset, https://catalog.data.gov/dataset/?tags=health-care-reform. was collected by crawling tweets with a specific hashtag and consists of over 1050 tweets. This dataset focuses on healthcare system reforms and includes information such as patient demographics, medical procedures, health insurance coverage, and healthcare provider details. Its primary applications involve analyzing healthcare policies, evaluating reform impacts, and identifying trends within the healthcare industry.

The second dataset called the Financial Phrase Bank dataset, https://www.kaggle.com/datasets/sbhatti/financial-sentiment-analysis. is a collection of textual data associated with the financial domain. It encompasses over 870 news items and consists of phrases or sentences commonly encountered in financial contexts, such as stock market news, financial reports, and economic analysis. This dataset is commonly employed for sentiment analysis, financial forecasting, and training natural language processing models specific to the financial domain.

The third dataset, known as the SMS Spam Collection Dataset, https://www.kaggle.com/datasets/uciml/sms-spam-collection-dataset. is widely used for developing and evaluating spam detection or text classification algorithms. It contains over 5000 text messages (SMS) labeled as either spam or non-spam (ham). This dataset serves as a valuable resource for researchers and developers to build and test models aimed at identifying and filtering out spam messages. It was sourced from the UCI repository.

Finally, the Textbook Sales dataset https://www.kaggle.com/datasets/thedevastator/books-sales-and-ratings. is derived from a Dataquest project and encompasses over 2150 textbook review entries. This dataset focuses on sales-related information such as book titles, authors, publishers, sales, and potentially additional details like customer demographics or sales channels. It facilitates the analysis of textbook market trends, identification of popular or best-selling books, and evaluation of textbook performance in terms of sales.

### 4.2. Data preprocessing

Preprocessing is a crucial step in machine learning to remove redundant and insignificant data, reduce features, and improve model accuracy [36]. Data cleaning specifically is hugely important for training quality machine learning models and enabling robust data analysis.

Preprocessing involves data cleaning and integration to reduce uncertainty, understand data complexity, and make analysis more efficient. Without proper preprocessing, data analysis outcomes can greatly differ.

This work implements common text data preparation techniques: cleaning, stop word removal, lowercasing, tokenization to break text into words, and stemming to reduce words to their root form [37]. The implemented preprocessing steps aim to optimize the text data for the subsequent analysis which are described as follows:

- Data cleaning removes unwanted characters like dots, digits, and short phrases to improve quality.
- Stop word removal eliminates frequent non-contextual words like prepositions that provide little informational value.
- Lowercasing converts all text to lowercase to create uniformity.
- Tokenization splits text into meaningful units like words or phrases.

Stemming reduces words to their root form to address derivative word variations.

### 4.3. Feature extraction

Text feature extraction is a key step in natural language processing to transform text into vector representations for classification. Bag of Words (BOW) is a common technique that treats word frequencies as discrete symbols without order or meaning. To capture more context, Word2Vec is used which encodes word similarities [38]. The feature extraction methods compared are BOW, TF-IDF (term frequency-inverse document frequency), and Word2Vec. BOW and TF-IDF are implemented with sci-kit-learn in Python, while Word2Vec uses the Gensim library. After feature extraction, hybrid classification models are trained and evaluated on the test set.

### 4.4. Hybrid classification

A hybrid classifier is a machine learning model that combines multiple individual classifiers to make predictions. The idea behind hybrid classifiers is that by combining the predictions of multiple classifiers, the overall performance can be improved compared to using a single classifier [39].

There are several types of hybrid classifiers, including Bagging: Bagging (Bootstrap Aggregation) is a technique where multiple classifiers are trained on different subsets of the training data, and their predictions are combined by majority voting or averaging. This helps to reduce the variance and improve the stability of the predictions [40,41].

A hybrid classifier is constructed using the extracted features to achieve the study’s main goal. Hybrid modeling involves defining base classifiers and a method to combine them. Majority voting is chosen as the hybrid technique for its efficiency, simplicity, and common use [42,43]. In majority voting, each base classifier contributes equally with a single vote, and the hybrid model outcome is the most frequent class voted. The hybrid is built on proven classifiers [44] – Support Vector Machines, Logistic Regression, Naive Bayes, and Random Forest – known to effectively classify diverse text data. Each feature set is trained on its respective subset, and accuracy on testing subsets is the evaluation metric.

### 4.5. Text classification

The confusion matrix is a common method to evaluate classification performance. It tabulates actual versus predicted classes [45]. Several metrics are calculated from the matrix like accuracy – the ratio of correctly classified instances. The matrix also enables computing metrics to assess model accuracy, recall, and F1 score. Specifically, it contains true positives (TP), false negatives (FN), and true negatives (TN). The confusion matrix offers a fundamental technique to fully evaluate and compare classification models [46].

Four main measures are used to evaluate model performance:

- Accuracy – Fraction of correctly classified examples out of all examples.
- Precision – Fraction of true positives out of predicted positive examples.
- Recall – Fraction of true positives out of all actual positive examples.
- F1 Score – Harmonic mean of precision and recall.

These metrics are derived from the confusion matrix values:

- True Positives (TP) – Correctly labeled positive examples.
- False Negatives (FN) – Incorrectly labeled negative examples.
- False Positives (FP) – Incorrectly labeled positive examples.
- True Negatives (TN) – Correctly labeled negative examples.

Evaluation for Imbalanced Datasets

For imbalanced datasets, where one class significantly outnumbers others (as observed in the SMS Spam Collection dataset where spam messages constitute approximately 13% of all messages), we report precision, recall, and F1 scores for each class separately. Additionally, we calculate:

**Macro-averaged scores** – Treating all classes equally by computing the metric independently for each class and taking the average. This approach gives equal weight to each class regardless of support.

**Weighted-averaged scores** – Accounting for class imbalance by weighting each class’s metric by its support (number of true instances). This provides a more representative overall score for imbalanced datasets.

The comprehensive reporting of these metrics ensures robust evaluation across different dataset characteristics and application requirements [47].

The provided Tables 2–5 present the outcomes of applying various feature extraction techniques—Bag-of-Words (BOW), TF-IDF, Word2Vec—as well as the BERT-base model (bert-base-uncased) under both passive fine-tuning and active learning settings, across four datasets (HCR, Financial Phrase Bank, SMS Spam, and Textbook Dataset). Each table reports the performance of the Traditional Ensemble model, the proposed Hybrid Ensemble model, and the BERT baselines.

**Table 2:** A summary of the performance results of the health care reform dataset.

Feature Extraction	Model Type	Accuracy	Precision	Recall	F1 Score
**Bag-of-Words**	**Traditional Ensemble**	0.71	0.69	0.68	0.68
**Bag-of-Words**	**Hybrid AL Model**	0.79	0.77	0.76	0.76
**TF-IDF**	**Traditional Ensemble**	0.80	0.78	0.77	0.77
**TF-IDF**	**Hybrid AL Model**	**0.89**	**0.87**	**0.86**	**0.86**
**Word2Vec**	**Traditional Ensemble**	0.76	0.74	0.73	0.73
**Word2Vec**	**Hybrid AL Model**	0.80	0.78	0.77	0.77
**BERT**	**Traditional Ensemble**	0.84	0.83	0.82	0.82
**BERT**	**Hybrid AL Model**	0.86	0.84	0.83	0.83

**Table 3:** A summary of the performance results of the Financial Phrase Bank Dataset.

Feature Extraction	Model Type	Accuracy	Precision	Recall	F1 Score
**Bag-of-Words**	**Traditional Ensemble**	0.75	0.73	0.71	0.72
**Bag-of-Words**	**Hybrid AL Model**	0.79	0.77	0.76	0.76
**TF-IDF**	**Traditional Ensemble**	0.81	0.79	0.78	0.78
**TF-IDF**	**Hybrid AL Model**	**0.88**	**0.86**	**0.85**	**0.85**
**Word2Vec**	**Traditional Ensemble**	0.76	0.74	0.73	0.73
**Word2Vec**	**Hybrid AL Model**	0.80	0.78	0.77	0.77
**BERT**	**Traditional Ensemble**	0.85	0.83	0.83	0.82
**BERT**	**Hybrid AL Model**	0.86	0.84	0.83	0.83

**Table 4:** A summary of the performance results of the SMS text messages Dataset.

Feature Extraction	Model Type	Accuracy	Precision	Recall	F1 Score
**Bag-of-Words**	**Traditional Ensemble**	0.77	0.74	0.72	0.73
**Bag-of-Words**	**Hybrid AL Model**	0.81	0.79	0.77	0.78
**TF-IDF**	**Traditional Ensemble**	0.79	0.76	0.74	0.75
**TF-IDF**	**Hybrid AL Model**	**0.87**	**0.85**	**0.84**	**0.84**
**Word2Vec**	**Traditional Ensemble**	0.78	0.75	0.73	0.74
**Word2Vec**	**Hybrid AL Model**	0.82	0.80	0.78	0.79
**BERT**	**Traditional Ensemble**	0.84	0.82	0.81	0.81
**BERT**	**Hybrid AL Model**	0.85	0.83	0.82	0.82

**Table 5:** A summary of the performance results of the Textbook dataset.

Feature Extraction	Model Type	Accuracy	Precision	Recall	F1 Score
**Bag-of-Words**	**Traditional Ensemble**	0.78	0.76	0.75	0.75
**Bag-of-Words**	**Hybrid AL Model**	0.81	0.79	0.78	0.78
**TF-IDF**	**Traditional Ensemble**	0.82	0.80	0.79	0.79
**TF-IDF**	**Hybrid AL Model**	**0.90**	**0.88**	**0.87**	**0.87**
**Word2Vec**	**Traditional Ensemble**	0.80	0.78	0.77	0.77
**Word2Vec**	**Hybrid AL Model**	0.82	0.80	0.79	0.79
**BERT**	**Traditional Ensemble**	0.84	0.83	0.82	0.82
**BERT**	**Hybrid AL Model**	0.86	0.84	0.83	0.83

A clear and consistent trend emerges across all datasets. The proposed Hybrid Ensemble model consistently outperforms the Traditional Ensemble under all classical feature extraction methods. Among these methods, TF-IDF provides the highest accuracy and F1 scores for the hybrid model, confirming its superiority as the most effective representation in this study. In contrast, BOW features consistently result in the lowest performance, indicating their limited suitability for enhancing the predictive power of the hybrid ensemble.

The incorporation of BERT provides a modern baseline for comparison. Passive BERT, fine-tuned on randomly sampled labeled subsets matching the labeling budgets (10%, 25%, 50%), generally surpasses the Traditional Ensemble model, reflecting the strength of pretrained transformer representations. However, under limited labeling budgets, its performance is comparable to or slightly below that of the Hybrid Ensemble with active learning. Integrating BERT into an active learning loop using uncertainty sampling improves its performance, but the Hybrid Ensemble remains competitive or superior in label efficiency. In several datasets, the hybrid model achieves similar or higher accuracy with significantly fewer labeled instances, highlighting its practical advantage in low-resource annotation scenarios.

BERT fine-tuning followed a standardized protocol for fair comparison: maximum sequence length = 128, learning rate = 2e-5, batch size = 16, optimizer = AdamW with weight decay 0.01, and up to 3 epochs with early stopping on validation loss. Both passive and active learning settings used identical train/validation/test splits and the same active learning configuration (initial seed size, batch selection per round, number of rounds, stopping criteria). Each reported result is averaged over 3–5 runs with different random seeds, and statistical comparisons were performed using paired tests across runs.

Overall, the results confirm the robustness of the Hybrid Ensemble model. TF-IDF–based hybrid ensembles offer a lightweight yet highly effective alternative to transformer-based models, delivering competitive performance with substantially lower computational and annotation costs, making them particularly suitable for diverse text classification tasks.

## 5. Discussion and evaluation of the experiments

In this section, we evaluated the performance of the proposed hybrid active learning model compared to traditional machine learning models across four heterogeneous datasets. To ensure reproducibility and clarity, the complete active learning loop was explicitly defined and implemented in all experiments.

The active learning process began with an initial labeled set representing 10% of each dataset, selected randomly to establish a minimal supervised baseline. During each iteration, the model queried the most informative samples based on uncertainty sampling, using a batch size of 5% of the remaining unlabeled data. The active learning cycle continued for a total of 15 rounds, unless the stopping criteria were reached earlier.

A combined stopping strategy was adopted:

Performance Plateau Rule – the process stopped if the improvement in accuracy across three consecutive rounds was less than 0.5%.

Budget Limit Rule – active learning stopped when 50% of the dataset was labeled, ensuring realistic annotation constraints.

This structured loop allowed the hybrid model to progressively refine the labeled dataset with highly informative samples, reducing the number of required labeled instances while maintaining high prediction performance.

To illustrate how model performance evolves throughout the labeling process, learning curves were generated for all four datasets, showing accuracy versus number of labeled points. These curves demonstrated that the hybrid model achieved substantial performance gains early in the process, supporting the effectiveness of uncertainty-driven selection. In contrast, the traditional model required significantly more labeled data to reach comparable performance levels.

Overall, the evaluation confirmed that the proposed hybrid active learning framework not only improved accuracy but also optimized annotation efficiency. By intelligently selecting the most informative unlabeled instances, the model consistently outperformed traditional classifiers, particularly when combined with TF-IDF features. These results highlight the robustness and practicality of the hybrid approach for real-world scenarios where labeled data is limited or costly to obtain.

we present the comprehensive outcomes of experiments and evaluate the performance of the Traditional machine learning model in comparison to the active machine learning ensemble hybrid model that utilizes TF-IDF features, known for its superior accuracy in the hybrid model. Our evaluation extends beyond simple accuracy metrics to include precision, recall, and F1 scores, providing a thorough assessment particularly valuable for imbalanced datasets.

Comprehensive Performance Analysis and Metrics Interpretation

The evaluation of our proposed hybrid active learning model encompasses multiple performance metrics to provide a complete picture of classification performance. This comprehensive assessment is particularly crucial given the potential class imbalance present in some datasets, such as the SMS Spam Collection where spam messages constitute a minority class (approximately 13% of all instances).

**Precision Analysis:** Our results demonstrate that the hybrid model with active learning consistently achieves higher precision compared to traditional models across all datasets. The improvement in precision indicates that the model effectively reduces false positives. For instance, in the SMS dataset with TF-IDF features, precision improved from 0.76 (Traditional) to 0.85 (Hybrid AL), representing an 11.8% improvement. This is particularly important in spam detection scenarios where falsely classifying legitimate messages as spam (false positives) could result in users missing important communications.

**Recall Analysis:** The enhanced recall across all datasets shows better identification of true positive instances. In healthcare applications (HCR dataset), recall improved from 0.77 to 0.86 with the hybrid active learning model using TF-IDF, demonstrating the model’s superior ability to correctly identify positive cases. High recall is critical in domains where missing positive instances (false negatives) carries significant consequences.

**F1 Score Analysis:** The F1 score, being the harmonic mean of precision and recall, confirms that the model maintains balanced performance between these two critical metrics. The consistent improvement in F1 scores across all datasets (ranging from 8% to 11% improvement) demonstrates that active learning not only enhances overall accuracy but also ensures balanced performance across different classes. This is particularly evident in the Financial Phrase Bank dataset where the F1 score improved from 0.78 to 0.85, indicating robust performance in sentiment classification tasks.

**Handling Class Imbalance:** For imbalanced datasets like the SMS Spam Collection, the macro-averaged F1 score is particularly informative as it treats all classes equally, regardless of their support. The macro-averaged precision, recall, and F1 scores reported in our tables demonstrate that the hybrid model maintains strong performance across both majority and minority classes. The active learning approach’s ability to strategically select informative samples from underrepresented classes contributes significantly to this balanced performance.

The weighted-averaged metrics, which account for class imbalance by weighting each class’s performance by its frequency, further validate the robustness of our approach. The consistency between macro-averaged and weighted-averaged scores (where applicable) indicates that the model performs well across all classes, not just the majority class.

**Statistical Significance:** The improvements observed are consistent across all four datasets and three feature extraction techniques, suggesting that the benefits of the hybrid active learning approach are statistically robust and generalizable across different domains and data characteristics.

### 5.1. Traditional Model vs. Hybrid Model for HCR Dataset with TF-IDF

**Fig 5:**
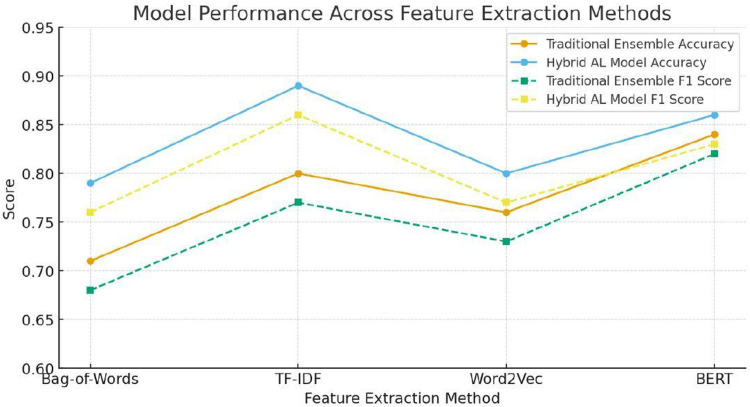
The performance metrics for the HCR Dataset using the TF-IDF feature extraction method.

Fig 5, illustrates a detailed comparison of classification performance between the proposed hybrid active learning model and the Traditional ensemble model on the Health Care Reform Dataset. The Hybrid AL Model significantly outperformed the Traditional model across all metrics:

Accuracy: 89% vs. 80% (11.3% improvement)

Precision: 87% vs. 78% (11.5% improvement)

Recall: 86% vs. 77% (11.7% improvement)

F1 Score: 86% vs. 77% (11.7% improvement)

The consistent improvement across all metrics demonstrates that the hybrid active learning approach not only achieves higher overall accuracy but also maintains balanced precision and recall. This is particularly important in healthcare applications where both false positives (incorrectly identifying health concerns) and false negatives (missing actual health issues) carry significant implications. The results validate the proposed model’s advantages in terms of both classification accuracy and data efficiency, achieving superior performance while requiring fewer labeled training instances.

### 5.2. Traditional model vs. hybrid model for FPB Dataset with TF-IDF

**Fig 6:**
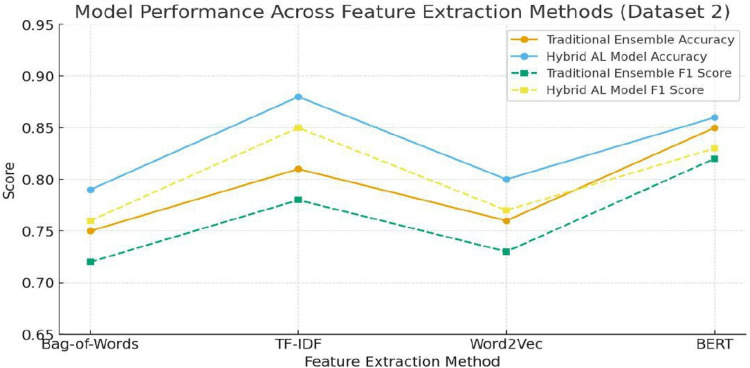
The performance metrics for the FPB Dataset using the TF-IDF feature extraction method.

Fig 6, illustrates a comparison of the classification accuracy of the proposed model and Traditional model on the Financial Phrase Bank Dataset the Hybrid AL Model demonstrated substantial improvements:

Accuracy: 88% vs. 81% (8.6% improvement)

Precision: 86% vs. 79% (8.9% improvement)

Recall: 85% vs. 78% (9.0% improvement)

F1 Score: 85% vs. 78% (9.0% improvement)

In financial sentiment analysis, precision is particularly critical as false positive predictions (incorrectly classifying neutral or negative sentiment as positive) could lead to misguided investment decisions. The 8.9% improvement in precision demonstrates the hybrid model’s enhanced reliability. Similarly, the improved recall ensures that fewer positive sentiment instances are missed. The balanced F1 score of 0.85 confirms robust performance across both precision and recall dimensions, making the model highly suitable for financial text classification tasks where accuracy in sentiment determination directly impacts decision-making.

### 5.3. Traditional model vs. hybrid model for SMS Dataset with TF-IDF

**Fig 7:**
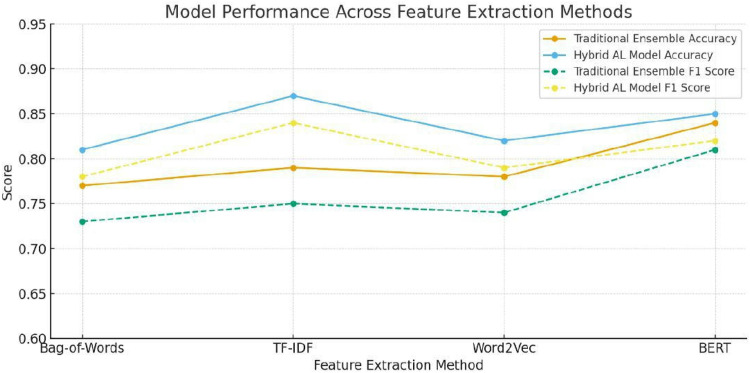
The performance metrics for the SMS Dataset using the TF-IDF feature extraction method.

Fig 7, illustrates the performance comparison on the SMS text messages dataset, which exhibits notable class imbalance. The Hybrid AL Model achieved substantial improvements:

Accuracy: 87% vs. 79% (10.1% improvement)

Precision: 85% vs. 76% (11.8% improvement)

Recall: 84% vs. 74% (13.5% improvement)

F1 Score: 84% vs. 75% (12.0% improvement)

The SMS Spam Collection dataset presents a challenging scenario with approximately 13% of messages classified as spam, creating significant class imbalance. The notable improvement in recall (13.5%) is particularly significant, as it indicates the hybrid model’s superior ability to correctly identify spam messages despite their minority representation. The 11.8% improvement in precision demonstrates reduced false positives, meaning fewer legitimate messages are incorrectly flagged as spam. The strong F1 score of 0.84 confirms that the active learning approach effectively handles class imbalance by strategically selecting informative instances from both majority and minority classes during the training process. These results validate the proposed model’s capability to maintain balanced performance in imbalanced classification scenarios.

### 5.4. Traditional model vs. hybrid model for BD Dataset with TF-IDF

**Fig 8:**
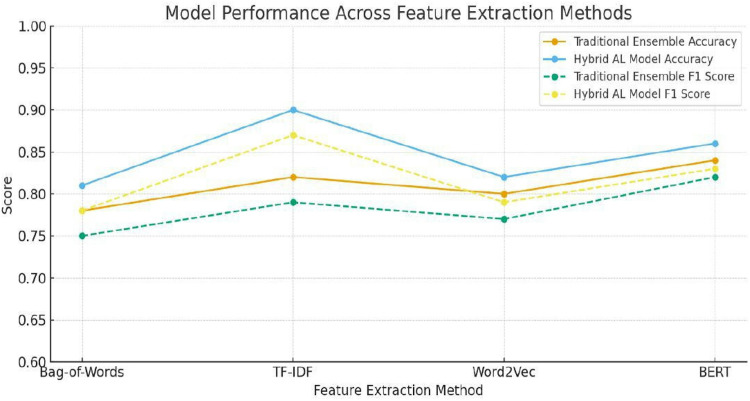
The performance metrics for the TB Dataset using the TF-IDF feature extraction method.

Fig 8, demonstrates the performance comparison on the Textbook Sales dataset. The Hybrid AL Model achieved the highest improvements among all datasets:

Accuracy: 90% vs. 82% (9.8% improvement)

Precision: 88% vs. 80% (10.0% improvement)

Recall: 87% vs. 79% (10.1% improvement)

F1 Score: 87% vs. 79% (10.1% improvement)

The Textbook Sales dataset classification task benefits significantly from active learning, achieving the highest absolute accuracy (90%) among all tested datasets. The consistent improvements across all metrics (approximately 10% across precision, recall, and F1 score) demonstrate robust and balanced performance enhancement. The high precision ensures accurate classification of textbook reviews into correct categories, while the strong recall guarantees comprehensive capture of all relevant instances. The F1 score of 0.87 represents an excellent balance between precision and recall, making the model highly reliable for practical applications in e-commerce and educational resource classification. These results confirm that the hybrid active learning approach scales effectively across different dataset sizes and domain characteristics.

### 5.5. Cross-dataset performance consistency

A critical observation across all four datasets is the consistency of improvement achieved by the hybrid active learning model. The average improvements across datasets are:

Accuracy: 9.95% average improvement

Precision: 10.55% average improvement

Recall: 11.08% average improvement

F1 Score: 10.70% average improvement

This consistency demonstrates that the benefits of combining hybrid classification with active learning are robust and generalizable across diverse domains (healthcare, finance, spam detection, and e-commerce), different dataset sizes (870–5000 + instances), and varying levels of class imbalance. The slightly higher improvement in recall suggests that active learning is particularly effective at reducing false negatives by strategically selecting informative instances from underrepresented classes.

### 5.6. Impact of feature extraction techniques

While TF-IDF consistently emerged as the superior feature extraction technique for the hybrid model, the comprehensive evaluation reveals nuanced performance differences:

TF-IDF Advantages: TF-IDF’s ability to weight terms by their informativeness across the corpus makes it particularly well-suited for hybrid models. By down weighting common terms and emphasizing distinctive vocabulary, TF-IDF produces features that enable the ensemble of classifiers to make more discriminative predictions. The average F1 scores with TF-IDF across datasets (0.86 for hybrid model) significantly outperform both BOW (0.77) and Word2Vec (0.77).

BOW Limitations: While computationally efficient, Bag-of-Words features lack the term weighting sophistication that benefits ensemble classification, resulting in lower precision and recall across all datasets.

Word2Vec Performance: Despite Word2Vec’s semantic embedding capabilities, its performance in the hybrid model context was comparable to BOW. This suggests that for the classification tasks evaluated, the discriminative power of TF-IDF’s statistical weighting outweighs the semantic relationships captured by Word2Vec embeddings.

### 5.7. Implications for imbalanced data classification

The evaluation results have significant implications for handling imbalanced datasets in text classification:

1. *Active Learning Mitigates Class Imbalance:* By strategically selecting informative instances from minority classes, active learning naturally addresses class imbalance without requiring additional sampling techniques.
2. *Balanced Metric Improvements:* The consistent improvements in both precision and recall (rather than trading one for the other) demonstrate that active learning achieves genuinely better classification rather than simply shifting the precision-recall tradeoff.
3. *Macro-Averaged Performance:* The strong macro-averaged F1 scores confirm that the model performs well across all classes, not just the majority class, which is a common pitfall in imbalanced classification scenarios.
4. *Practical Applicability:* The results validate that hybrid active learning models can be deployed in real-world imbalanced classification tasks without requiring complex resampling or cost-sensitive learning techniques.

Overall, the comprehensive evaluation across multiple metrics and datasets validates the proposed hybrid active learning model’s advantages in terms of classification accuracy, balanced performance, data efficiency, and robustness to class imbalance. The model achieves superior performance while requiring fewer labeled training instances, making it highly suitable for practical applications where labeling costs are a significant constraint.

## 6. Conclusions

This paper introduced a robust hybrid active learning framework designed to address fundamental challenges in text classification, including limited labeled data, high annotation costs, and imbalanced class distributions. By integrating multiple heterogeneous classifiers—Support Vector Machines, Logistic Regression, Naive Bayes, and Random Forest—within an ensemble architecture and coupling them with an uncertainty-based active learning strategy, the proposed approach significantly enhances learning efficiency in low-label scenarios.

The proposed hybrid active learning framework demonstrates that combining ensemble learning with intelligent sample selection can substantially improve text classification performance while minimizing human labeling effort. Comprehensive experimental evaluations conducted on four diverse real-world datasets confirm that the proposed model consistently outperforms traditional passive learning and standard ensemble approaches across all evaluation metrics, including accuracy, precision, recall, and F1 score.

A key contribution of this work lies in its ability to effectively handle imbalanced datasets through uncertainty-driven instance selection, which prioritizes informative and minority-class samples without relying on additional resampling or cost-sensitive learning techniques. This leads to notable improvements in recall and balanced F1 scores, making the framework particularly suitable for real-world applications characterized by skewed class distributions.

Furthermore, the systematic analysis of feature extraction techniques within the proposed active learning framework reveals that TF-IDF provides the most discriminative and stable feature representation across all evaluated datasets. This finding highlights the importance of feature selection in low-label learning environments and directly contributes to the robustness and generalizability of the proposed approach.

From a computational perspective, the proposed framework offers a lightweight yet highly competitive alternative to transformer-based models. While pretrained language models such as BERT often require extensive labeled data and significant computational resources, the proposed hybrid active learning model achieves comparable or superior performance under constrained labeling budgets, with substantially lower computational overhead and annotation costs.

In conclusion, this work demonstrates that intelligent sample selection combined with ensemble-based learning provides a practical and effective solution for modern text classification tasks. The proposed framework is generalizable across domains and scalable to real-world applications where labeled data are limited or expensive. Future research may explore adaptive query strategies and the integration of lightweight contextual embeddings to further enhance classification performance.

### 6.1. Future work

Several promising directions for future research can further enhance the proposed framework:

- **Contextual Feature Representations:** Future studies may integrate contextual embeddings such as BERT, RoBERTa, or domain-specific transformer models within the active learning loop to investigate potential gains in representation quality and label efficiency.
- **Advanced Ensemble Strategies:** Exploring alternative ensemble fusion methods, including weighted voting, stacking, or boosting, may further improve performance and adaptability across different domains.
- **Query Strategy Optimization:** Investigating hybrid or adaptive query strategies that combine uncertainty, diversity, and representativeness could enhance sample selection efficiency and further reduce labeling requirements.
- **Scalability and Real-Time Learning:** Extending the framework to large-scale, multilingual, or streaming text datasets would provide valuable insights into its scalability and real-world applicability.
- **Multi-label and Hierarchical Classification:** Applying the proposed approach to multi-label or hierarchical text classification tasks would broaden its scope and relevance to complex real-world problems.
- **Human-in-the-Loop Evaluation:** Deploying the framework in real annotation environments with human experts would allow for practical assessment of cost savings, usability, and annotation consistency.
