# Supplementary material for: A novel hybrid model for identifying the most informative instances for improving text data classification
Source: PLoS One. 2026 Aug 10;21(8):e0355603. doi: 10.1371/journal.pone.0355603 (PMC13456273; doi:10.1371/journal.pone.0355603)
Supplement: Supplementary file 1 [file pone.0355603.s002.docx]

**Response to Reviewers**

**Manuscript ID:** PONE-D-25-44944
**Title:** *A Novel Hybrid Model for Identifying the Most Informative Instances for Improving Text Data Classification*

Dear Academic Editor and Reviewers,

We would like to sincerely thank the Academic Editor and the reviewers for their careful evaluation of our manuscript and for their constructive and insightful comments. We greatly appreciate the time and effort devoted to reviewing our work. The reviewers’ suggestions were highly valuable and significantly contributed to improving the clarity, rigor, and overall quality of the manuscript.

We are pleased to report that **all comments and required revisions have been fully addressed**. The manuscript has been carefully revised, expanded, and improved accordingly. Below, we provide a detailed point-by-point response to each reviewer’s comment, indicating how the manuscript has been revised.

**Response to Reviewer #1**

**General Comment**

We thank the reviewer for recognizing the practical relevance of the proposed hybrid active learning framework and its effectiveness for label-efficient text classification.

**Comment 1: Baseline ambiguity between traditional ensemble and hybrid ensemble**

**Response:**
We agree with the reviewer and have clarified this important distinction. The manuscript now explicitly explains that the *Traditional Ensemble* uses passive learning with randomly selected labeled samples, while the *Hybrid Ensemble* integrates the same base classifiers within a **pool-based active learning loop using uncertainty sampling**. This clarification has been added in Section **5.4, Page 10, second and fifth paragraphs** after Tables (2-5) which reports the performance of the Traditional Ensemble model, the proposed Hybrid Ensemble model, and the BERT baselines.

“A clear and consistent trend emerges across all datasets. The proposed Hybrid Ensemble model consistently outperforms the Traditional Ensemble under all classical feature extraction methods. Among these methods, TF-IDF provides the highest accuracy and F1 scores for the hybrid model, confirming its superiority as the most effective representation in this study. In contrast, BOW features consistently result in the lowest performance, indicating their limited suitability for enhancing the predictive power of the hybrid ensemble.”

“Overall, the results confirm the robustness of the Hybrid Ensemble model. TF-IDF–based hybrid ensembles offer a lightweight yet highly effective alternative to transformer-based models, delivering competitive performance with substantially lower computational and annotation costs, making them particularly suitable for diverse text classification tasks.”

**Comment 2: Specify active learning loop details**

**Response:**
This comment has been fully addressed. We have explicitly described:

- Initial labeled set size (10%)
- Batch size per iteration (5%)
- Number of rounds (up to 15)
- Stopping criteria (performance plateau and labeling budget limit)

These details are now clearly presented in:

**Sections 4.0 (Methodology):** Second paragraph - complete description of pool-based active learning approach**.**

**“**The research utilizes a pool-based active learning approach. Unlabeled data is divided into batches that are selectively sampled for labeling by a human expert. After each batch is labeled, the algorithm is retrained on the labeled data. This iterative process of sampling, labeling, and retraining continues until model accuracy reaches an acceptable level. For sample selection, an uncertainty sampling method is used to choose the most informative instances to label from the unlabeled data pool. After each round of labeling, model accuracy is evaluated to determine if additional labeling is needed. Once sufficient accuracy is achieved, the final labeled dataset is used to train a classifier.”

**Section 5.0**. **(Discussion):** First three paragraphs describing the active learning process, stopping criteria, and implementation details.

“The active learning process began with an initial labeled set representing 10% of each dataset, selected randomly to establish a minimal supervised baseline. During each iteration, the model queried the most informative samples based on uncertainty sampling, using a batch size of 5% of the remaining unlabeled data. The active learning cycle continued for a total of 15 rounds, unless the stopping criteria were reached earlier.

A combined stopping strategy was adopted:

Performance Plateau Rule – the process stopped if the improvement in accuracy across three consecutive rounds was less than 0.5%.

Budget Limit Rule – active learning stopped when 50% of the dataset was labeled, ensuring realistic annotation constraints.”

Additionally, learning curves illustrating accuracy versus number of labeled samples have been generated and discussed to demonstrate label efficiency.

**Comment 3: Report precision, recall, and F1 for imbalanced datasets**

**Response:**
We have expanded the evaluation to include **precision, recall, and F1-score** for all datasets, with special emphasis on imbalanced data (SMS Spam dataset). Macro-averaged and weighted metrics are now reported and discussed in **Section 4.5 and Section 5.0**, providing a comprehensive evaluation.

**Modification Locations in Manuscript:**

- **Abstract:** Mentioning "accuracy, precision, recall, and F1 score" and "imbalanced classification scenarios"

“In particular, TF-IDF-based hybrid ensembles achieve the highest gains in accuracy, precision, recall, and F1 score, while requiring substantially fewer labeled instances. Furthermore, the proposed framework exhibits strong robustness in imbalanced classification scenarios, significantly improving minority class detection.”

- **Section 4.5 (Text Classification):** Completely new subsection titled "Evaluation for Imbalanced Datasets"

**“Evaluation for Imbalanced Datasets**

For imbalanced datasets, where one class significantly outnumbers others (as observed in the SMS Spam Collection dataset where spam messages constitute approximately 13% of all messages), we report precision, recall, and F1 scores for each class separately. Additionally, we calculate:

**Macro-averaged scores** - Treating all classes equally by computing the metric independently for each class and taking the average. This approach gives equal weight to each class regardless of support.

**Weighted-averaged scores** - Accounting for class imbalance by weighting each class's metric by its support (number of true instances). This provides a more representative overall score for imbalanced datasets.

The comprehensive reporting of these metrics ensures robust evaluation across different dataset characteristics and application requirements [47].”

- **Section 4.5: in Tables 2-5:** columns are added for Precision, Recall, and F1 Score for all models
- **Section 5.0:** New comprehensive section "Comprehensive Performance Analysis and Metrics Interpretation" with detailed analysis of Precision, Recall, and F1

**“Comprehensive Performance Analysis and Metrics Interpretation**

The evaluation of our proposed hybrid active learning model encompasses multiple performance metrics to provide a complete picture of classification performance. This comprehensive assessment is particularly crucial given the potential class imbalance present in some datasets, such as the SMS Spam Collection where spam messages constitute a minority class (approximately 13% of all instances).

**Precision Analysis:** Our results demonstrate that the hybrid model with active learning consistently achieves higher precision compared to traditional models across all datasets. The improvement in precision indicates that the model effectively reduces false positives. For instance, in the SMS dataset with TF-IDF features, precision improved from 0.76 (Traditional) to 0.85 (Hybrid AL), representing an 11.8% improvement. This is particularly important in spam detection scenarios where falsely classifying legitimate messages as spam (false positives) could result in users missing important communications.

**Recall Analysis:** The enhanced recall across all datasets shows better identification of true positive instances. In healthcare applications (HCR dataset), recall improved from 0.77 to 0.86 with the hybrid active learning model using TF-IDF, demonstrating the model's superior ability to correctly identify positive cases. High recall is critical in domains where missing positive instances (false negatives) carries significant consequences.

**F1 Score Analysis:** The F1 score, being the harmonic mean of precision and recall, confirms that the model maintains balanced performance between these two critical metrics. The consistent improvement in F1 scores across all datasets (ranging from 8% to 11% improvement) demonstrates that active learning not only enhances overall accuracy but also ensures balanced performance across different classes. This is particularly evident in the Financial Phrase Bank dataset where the F1 score improved from 0.78 to 0.85, indicating robust performance in sentiment classification tasks.”

- **Figures 5-8:** Updated bar charts showing all four metrics
- **Conclusions:** Emphasis on balanced performance and imbalanced dataset handling

“Beyond overall accuracy improvements, the proposed framework demonstrated strong robustness in handling imbalanced datasets. The active learning mechanism effectively prioritized informative instances from minority classes, leading to substantial gains in recall and balanced F1 scores without relying on additional resampling or cost-sensitive learning techniques.”

**Comment 4: Explicitly mention human annotation**

**Response:**
We have clarified that human annotation is simulated within the pool-based active learning framework, where a human oracle provides labels for the selected informative samples. This clarification has been added and described in **Section 3.1** and Figure 3.

**Comment 5: Benchmark against state-of-the-art methods including BERT**

**Response:**
We thank the reviewer for this valuable suggestion. We have added **BERT-based baselines** (passive fine-tuning and active learning) and provided a detailed comparison with the proposed hybrid model. Experimental settings and hyperparameters are fully described, and results are reported and discussed in **Section 4.5 and Tables 2–5**.

**Modification Locations in Manuscript:**

- **Abstract:** Added "BERT-based embeddings" and "transformer-based approaches" mentions
- **Tables 2-5:** Two new rows added for each table - "BERT Traditional Ensemble" and "BERT Hybrid AL Model"

The provided tables (2–5) present the outcomes of applying various feature extraction techniques—Bag-of-Words (BOW), TF-IDF, Word2Vec—as well as the BERT-base model (bert-base-uncased) under both passive fine-tuning and active learning settings, across four datasets (HCR, Financial Phrase Bank, SMS Spam, and Textbook Dataset). Each table reports the performance of the Traditional Ensemble model, the proposed Hybrid Ensemble model, and the BERT baselines.

- **After Tables (Section 4.5):** Complete paragraph describing BERT experimental protocol, hyperparameters, and comparison methodology

“The incorporation of BERT provides a modern baseline for comparison. Passive BERT, fine-tuned on randomly sampled labeled subsets matching the labeling budgets (10%, 25%, 50%), generally surpasses the Traditional Ensemble model, reflecting the strength of pretrained transformer representations. However, under limited labeling budgets, its performance is comparable to or slightly below that of the Hybrid Ensemble with active learning. Integrating BERT into an active learning loop using uncertainty sampling improves its performance, but the Hybrid Ensemble remains competitive or superior in label efficiency. In several datasets, the hybrid model achieves similar or higher accuracy with significantly fewer labeled instances, highlighting its practical advantage in low-resource annotation scenarios.

BERT fine-tuning followed a standardized protocol for fair comparison: maximum sequence length = 128, learning rate = 2e-5, batch size = 16, optimizer = AdamW with weight decay 0.01, and up to 3 epochs with early stopping on validation loss. Both passive and active learning settings used identical train/validation/test splits and the same active learning configuration (initial seed size, batch selection per round, number of rounds, stopping criteria). Each reported result is averaged over 3–5 runs with different random seeds, and statistical comparisons were performed using paired tests across runs.”

- **Conclusions:** Statement comparing computational efficiency and cost-effectiveness versus transformer-based approaches

“From a computational perspective, the proposed framework offers a lightweight yet highly competitive alternative to transformer-based models. While pretrained language models such as BERT often require extensive labeled data and significant computational resources, the proposed hybrid active learning model achieves comparable or superior performance under constrained labeling budgets, with substantially lower computational overhead and annotation costs.”

**Comment 6: Provide hyperparameters and implementation details**

**Response:**
All implementation details, including feature extraction settings, classifier configurations, active learning parameters, and BERT fine-tuning hyperparameters, have been explicitly reported in **Section 4.5** to ensure reproducibility.

Section 4.5 includes all implementation details Besides the following paragraph regarding BERT.

“BERT fine-tuning followed a standardized protocol for fair comparison: maximum sequence length = 128, learning rate = 2e-5, batch size = 16, optimizer = AdamW with weight decay 0.01, and up to 3 epochs with early stopping on validation loss. Both passive and active learning settings used identical train/validation/test splits and the same active learning configuration (initial seed size, batch selection per round, number of rounds, stopping criteria). Each reported result is averaged over 3–5 runs with different random seeds, and statistical comparisons were performed using paired tests across runs.”

**Additional Literature**

**Response:**
All recommended references have been carefully reviewed and incorporated into the Related Work section, including:

- Zafar et al. (2024)
- Ren et al. (2021)
- Schröder & Niekler (2020)
- Zhang et al. (2022)

These works are now properly cited and discussed in **Section 2.0 (Related Work)**.

**Modification Locations in Manuscript:**

- **Section 2.0 (Related Work):** Two new paragraphs added at the end of the section discussing:
  - Hybrid and attention-based architecture (Zafar et al., 2024)
  - Modern active learning strategies and query design (Ren et al., Schröder & Niekler, Zhang et al.)

“Hybrid models, which combine multiple classifiers, have gained considerable attention due to their ability to leverage the strengths of different learning algorithms and improve generalization performance [20,21]. Recent research trends further emphasize the effectiveness of hybrid and attention-based architectures. For example, attention-driven multi-branch hybrid designs have been shown to enhance robustness and efficiency by adaptively fusing information from multiple components. Although primarily explored in computer vision, such principles provide useful insights for text classification and reinforce the motivation for adopting hybrid classifier ensembles.

In parallel, modern studies on active learning provide comprehensive guidance for designing effective query strategies. Active learning techniques focus on uncertainty sampling, diversity-based selection, and hybrid strategies that combine multiple criteria to identify the most informative samples [22,23]. These approaches have proven particularly effective for text classification, where labeling costs are high and data distributions are complex. Empirical evidence indicates that active learning can significantly reduce annotation effort while maintaining or even improving classification accuracy compared to passive learning [24,25].”

- **References Section:** All four required citations properly added with full bibliographic details

**Response to Reviewer #2**

We sincerely thank Reviewer #2 for the detailed and constructive feedback, which greatly helped improve the manuscript.

**Comment: Clarify contribution and novelty**

**Response:**
We have refined the contribution statement in the **Introduction and Conclusions**, clearly highlighting the novelty of integrating a hybrid ensemble with an active learning loop and demonstrating its effectiveness under constrained labeling budgets.

**Modification Locations in Manuscript:**

- **Introduction**

“To address these challenges, this paper proposes a novel hybrid active learning framework for text classification that combines ensemble learning with uncertainty-based sample selection. Instead of relying on a single classifier, the proposed approach integrates multiple heterogeneous learning algorithms—Support Vector Machines, Logistic Regression, Naive Bayes, and Random Forest—within a unified ensemble architecture. This hybrid design exploits the complementary strengths of different classifiers, leading to improved robustness and generalization under constrained labeling budgets. Furthermore, the framework systematically evaluates multiple feature extraction techniques within an active learning setting and demonstrates that TF-IDF consistently provides superior and more stable performance across diverse datasets.

In addition to improving overall classification accuracy, the proposed framework effectively addresses class imbalance by prioritizing uncertain and informative instances, particularly from minority classes, during the active learning process. This strategy enhances recall and balanced F1 scores without requiring additional resampling or cost-sensitive learning techniques. Extensive experiments conducted on multiple real-world datasets from healthcare, financial sentiment analysis, spam detection, and e-commerce domains validate the effectiveness, efficiency, and generalizability of the proposed approach. The results further show that the proposed hybrid active learning framework offers a computationally efficient alternative to transformer-based models, achieving competitive or superior performance under limited labeling budgets with significantly lower computational and annotation costs.”

- **Conclusions**

“A key contribution of this work lies in its ability to effectively handle imbalanced datasets through uncertainty-driven instance selection, which prioritizes informative and minority-class samples without relying on additional resampling or cost-sensitive learning techniques. This leads to notable improvements in recall and balanced F1 scores, making the framework particularly suitable for real-world applications characterized by skewed class distributions.”

**Comment: Experimental setup details**

**Response:**
All experimental details—including dataset splits, batch sizes, number of rounds, stopping rules, random seeds, and hyperparameters—have now been clearly documented in **Sections 4.5 and 5.0**.

**Modification Locations in Manuscript:**

- **Section 4.5**

“BERT fine-tuning followed a standardized protocol for fair comparison: maximum sequence length = 128, learning rate = 2e-5, batch size = 16, optimizer = AdamW with weight decay 0.01, and up to 3 epochs with early stopping on validation loss. Both passive and active learning settings used identical train/validation/test splits and the same active learning configuration (initial seed size, batch selection per round, number of rounds, stopping criteria). Each reported result is averaged over 3–5 runs with different random seeds, and statistical comparisons were performed using paired tests across runs.”

- **Section 5.0, first three paragraphs**

“The active learning process began with an initial labeled set representing 10% of each dataset, selected randomly to establish a minimal supervised baseline. During each iteration, the model queried the most informative samples based on uncertainty sampling, using a batch size of 5% of the remaining unlabeled data. The active learning cycle continued for a total of 15 rounds, unless the stopping criteria were reached earlier.

A combined stopping strategy was adopted:

Performance Plateau Rule – the process stopped if the improvement in accuracy across three consecutive rounds was less than 0.5%.

Budget Limit Rule – active learning stopped when 50% of the dataset was labeled, ensuring realistic annotation constraints.”

**Comment: Show accuracy versus labeled sample size**

**Response:**
Learning curves have been generated and discussed in **Section 5.0**, demonstrating how the proposed hybrid active learning model achieves competitive or superior performance using fewer labeled samples.

**Modification Locations in Manuscript:**

- **Section 5.0 (Discussion):** Complete paragraph dedicated to learning curves analysis

“To illustrate how model performance evolves throughout the labeling process, learning curves were generated for all four datasets, showing accuracy versus number of labeled points. These curves demonstrated that the hybrid model achieved substantial performance gains early in the process, supporting the effectiveness of uncertainty-driven selection. In contrast, the traditional model required significantly more labeled data to reach comparable performance”

- **Sections 4.0 (Methodology):** Second paragraph - complete description of pool-based active learning approach**.**

“The research utilizes a pool-based active learning approach. Unlabeled data is divided into batches that are selectively sampled for labeling by a human expert. After each batch is labeled, the algorithm is retrained on the labeled data. This iterative process of sampling, labeling, and retraining continues until model accuracy reaches an acceptable level. For sample selection, an uncertainty sampling method is used to choose the most informative instances to label from the unlabeled data pool. After each round of labeling, model accuracy is evaluated to determine if additional labeling is needed. Once sufficient accuracy is achieved, the final labeled dataset is used to train a classifier.”

- **Section 5.0**. **(Discussion):** First three paragraphs describing the active learning process, stopping criteria, and implementation details.

“In this section, we evaluated the performance of the proposed hybrid active learning model compared to traditional machine learning models across four heterogeneous datasets. To ensure reproducibility and clarity, the complete active learning loop was explicitly defined and implemented in all experiments.

The active learning process began with an initial labeled set representing 10% of each dataset, selected randomly to establish a minimal supervised baseline. During each iteration, the model queried the most informative samples based on uncertainty sampling, using a batch size of 5% of the remaining unlabeled data. The active learning cycle continued for a total of 15 rounds, unless the stopping criteria were reached earlier.

A combined stopping strategy was adopted:

Performance Plateau Rule – the process stopped if the improvement in accuracy across three consecutive rounds was less than 0.5%.

Budget Limit Rule – active learning stopped when 50% of the dataset was labeled, ensuring realistic annotation constraints.”

Additionally, learning curves illustrating accuracy versus number of labeled samples have been generated and discussed to demonstrate label efficiency.

**Comment: Stronger evaluation of metrics and confusion matrices**

**Response:**
We now report **accuracy, precision, recall, F1-score**, and explicitly discuss performance on imbalanced datasets. Confusion matrix-based metrics are described in **Section 4.5**.

**Comment: Dataset transparency**

**Response:**
Dataset descriptions have been expanded in **Section 4.1**, including dataset sources, sizes, domains, and class distributions. Datasets links are added as well.

“We have gathered data from four different domains, which consist of datasets of different sizes and sources. These datasets are abbreviated as follows: HCR dataset (Health Care Reform), FPB dataset (Financial Phrase Bank), SSC dataset (SMS Spam Collection), and TBS dataset (Textbook Sales). In this subsection, we will provide a summary of each of these datasets.

The first dataset, known as the Health Care Reform dataset, <https://catalog.data.gov/dataset/?tags=health-care-reform>. was collected by crawling tweets with a specific hashtag and consists of over 1050 tweets. This dataset focuses on healthcare system reforms and includes information such as patient demographics, medical procedures, health insurance coverage, and healthcare provider details. Its primary applications involve analyzing healthcare policies, evaluating reform impacts, and identifying trends within the healthcare industry.

The second dataset called the Financial Phrase Bank dataset, <https://www.kaggle.com/datasets/sbhatti/financial-sentiment-analysis>. is a collection of textual data associated with the financial domain. It encompasses over 870 news items and consists of phrases or sentences commonly encountered in financial contexts, such as stock market news, financial reports, and economic analysis. This dataset is commonly employed for sentiment analysis, financial forecasting, and training natural language processing models specific to the financial domain.

The third dataset, known as the SMS Spam Collection Dataset, <https://www.kaggle.com/datasets/uciml/sms-spam-collection-dataset>. is widely used for developing and evaluating spam detection or text classification algorithms. It contains over 5000 text messages (SMS) labeled as either spam or non-spam (ham). This dataset serves as a valuable resource for researchers and developers to build and test models aimed at identifying and filtering out spam messages. It was sourced from the UCI repository.

Finally, the Textbook Sales dataset <https://www.kaggle.com/datasets/thedevastator/books-sales-and-ratings>. is derived from a Dataquest project and encompasses over 2150 textbook review entries. This dataset focuses on sales-related information such as book titles, authors, publishers, sales, and potentially additional details like customer demographics or sales channels. It facilitates the analysis of textbook market trends, identification of popular or best-selling books, and evaluation of textbook performance in terms of sales.”

**Comment: Improve writing and captions**

**Response:**
The manuscript has been carefully edited for clarity and language quality. Figure and table captions have been revised to clearly convey their intended insights.

**Editorial and Journal Requirements**

- All PLOS ONE formatting requirements have been followed.
- Tables 2–5 are now explicitly referenced in the manuscript text.
- The **Role of the Funder** statement has been clarified:
  *“The funders had no role in study design, data collection and analysis, decision to publish, or preparation of the manuscript.”*
- The **Data Availability Statement** has been reviewed and confirmed to include all minimal datasets required for result replication, The datasets URLs are added at the end of the manuscript.
- The funding statement is updated to “This work was supported by the Deanship of Scientific Research, Vice Presidency for Graduate Studies and Scientific Research, King Faisal University, Saudi Arabia [Grant No. 254526].

This update is also mentioned in the cover letter.

**Closing Statement**

Once again, we sincerely thank the Academic Editor and the reviewers for their constructive feedback. We believe that the revisions have significantly strengthened the manuscript in terms of clarity, methodological rigor, experimental completeness, and reproducibility. We are confident that the revised version now meets the publication criteria of **PLOS ONE**, and we hope it will be found suitable for publication.

Thank you very much for your time and consideration.

Kind regards,
**Amira Abdelwahab**
**Mohamed Salama**
