# Supplementary material for: A novel hybrid model for identifying the most informative instances for improving text data classification
Source: PLoS One. 2026 Aug 10;21(8):e0355603. doi: 10.1371/journal.pone.0355603 (PMC13456273; doi:10.1371/journal.pone.0355603)
Supplement: Peer review history

# Peer review: Decision Letter 0

**DOI:** 10.1371/journal.pone.0355603.r001

**Authors:** Divya Chaudhary

9 Dec 2025

Dear Dr. Abdelwahab,

Please submit your revised manuscript by Jan 23 2026 11:59PM. If you will need more time than this to complete your revisions, please reply to this message or contact the journal office at plosone@plos.org. When you're ready to submit your revision, log on to https://www.editorialmanager.com/pone/ and select the 'Submissions Needing Revision' folder to locate your manuscript file.

- A rebuttal letter that responds to each point raised by the academic editor and reviewer(s). You should upload this letter as a separate file labeled 'Response to Reviewers'.
- A marked-up copy of your manuscript that highlights changes made to the original version. You should upload this as a separate file labeled 'Revised Manuscript with Track Changes'.
- An unmarked version of your revised paper without tracked changes. You should upload this as a separate file labeled 'Manuscript'.

If you would like to make changes to your financial disclosure, please include your updated statement in your cover letter. Guidelines for resubmitting your figure files are available below the reviewer comments at the end of this letter.

If applicable, we recommend that you deposit your laboratory protocols in protocols.io to enhance the reproducibility of your results. Protocols.io assigns your protocol its own identifier (DOI) so that it can be cited independently in the future. For instructions see: https://journals.plos.org/plosone/s/submission-guidelines#loc-laboratory-protocols. Additionally, PLOS ONE offers an option for publishing peer-reviewed Lab Protocol articles, which describe protocols hosted on protocols.io. Read more information on sharing protocols at https://plos.org/protocols?utm_medium=editorial-email&utm_source=authorletters&utm_campaign=protocols.

We look forward to receiving your revised manuscript.

Kind regards,

Divya Chaudhary, Ph.D.

Academic Editor

PLOS One

Journal Requirements:

When submitting your revision, we need you to address these additional requirements.

1. Please ensure that your manuscript meets PLOS ONE's style requirements, including those for file naming. The PLOS ONE style templates can be found at

https://journals.plos.org/plosone/s/file?id=wjVg/PLOSOne_formatting_sample_main_body.pdf and

https://journals.plos.org/plosone/s/file?id=ba62/PLOSOne_formatting_sample_title_authors_affiliations.pdf

2. Please note that PLOS One has specific guidelines on code sharing for submissions in which author-generated code underpins the findings in the manuscript. In these cases, we expect all author-generated code to be made available without restrictions upon publication of the work. Please review our guidelines at https://journals.plos.org/plosone/s/materials-and-software-sharing#loc-sharing-code and ensure that your code is shared in a way that follows best practice and facilitates reproducibility and reuse.

3. Thank you for stating the following financial disclosure:

This work was supported through the Annual Funding track by the Deanship of Scientific Research, Vice Presidency for Graduate Studies and Scientific Research, King Faisal University, Saudi Arabia.

Please state what role the funders took in the study. If the funders had no role, please state: "The funders had no role in study design, data collection and analysis, decision to publish, or preparation of the manuscript."

If this statement is not correct you must amend it as needed.

Please include this amended Role of Funder statement in your cover letter; we will change the online submission form on your behalf.

4. We note that your Data Availability Statement is currently as follows: All relevant data are within the manuscript and its Supporting Information files

Please confirm at this time whether or not your submission contains all raw data required to replicate the results of your study. Authors must share the “minimal data set” for their submission. PLOS defines the minimal data set to consist of the data required to replicate all study findings reported in the article, as well as related metadata and methods (https://journals.plos.org/plosone/s/data-availability#loc-minimal-data-set-definition).

For example, authors should submit the following data:

- The values behind the means, standard deviations and other measures reported;

- The values used to build graphs;

- The points extracted from images for analysis.

Authors do not need to submit their entire data set if only a portion of the data was used in the reported study.

If your submission does not contain these data, please either upload them as Supporting Information files or deposit them to a stable, public repository and provide us with the relevant URLs, DOIs, or accession numbers. For a list of recommended repositories, please see https://journals.plos.org/plosone/s/recommended-repositories.

If there are ethical or legal restrictions on sharing a de-identified data set, please explain them in detail (e.g., data contain potentially sensitive information, data are owned by a third-party organization, etc.) and who has imposed them (e.g., an ethics committee). Please also provide contact information for a data access committee, ethics committee, or other institutional body to which data requests may be sent. If data are owned by a third party, please indicate how others may request data access.

5. We note you have included a table to which you do not refer in the text of your manuscript. Please ensure that you refer to Table 2-5 in your text; if accepted, production will need this reference to link the reader to the Tables.

6. If the reviewer comments include a recommendation to cite specific previously published works, please review and evaluate these publications to determine whether they are relevant and should be cited. There is no requirement to cite these works unless the editor has indicated otherwise.

Reviewers' comments:

Reviewer's Responses to Questions

**Comments to the Author**

1. Is the manuscript technically sound, and do the data support the conclusions?

Reviewer #1: Partly

Reviewer #2: Yes

**********

2. Has the statistical analysis been performed appropriately and rigorously?

Reviewer #1: No

Reviewer #2: No

**********

3. Have the authors made all data underlying the findings in their manuscript fully available?

The PLOS Data policy

Reviewer #1: No

Reviewer #2: Yes

**********

4. Is the manuscript presented in an intelligible fashion and written in standard English?

Reviewer #1: Yes

Reviewer #2: Yes

**********

Reviewer #1: Summary:

The manuscript proposes a pool based active learning workflow for text classification using uncertainty sampling to query labels and a simple majority vote ensemble of SVM, Logistic Regression, Naive Bayes, and Random Forest over TF IDF. It tests on four small datasets HCR, FPB, SMS Spam, and Textbook Sales, the hybrid with active approach outperforms the paper’s baseline model, with the largest gains when using TF IDF features. The idea is practical and relevant for label efficient text classification.

Required Revisions

- Baseline Ambiguity: The paper compares a traditional ensemble machine learning model to a hybrid ensemble machine learning model, they are both ensemble methods, can you please specify what is different?

- Specify Active Learning Loop: Please add more details regarding initial labeled set size, batch size, number of rounds, stopping rule values, and consider adding learning curves showing performance vs number of labeled points for each dataset.

- Report precision, recall, F1 scores as well specifically for imbalance datasets

- Please mention explicitly where human annotation was involved in the pool based settings during simulation

- Please benchmark against latest state of the art methods including BERT

- Provide all hyperparameters and implementation details for reproducibility

Please consider adding latest relevant literature

Related work on hybrid and attention ideas

Zafar et al., 2024; Single Stage Adaptive Multi‑Attention Network for Image Restoration (SSAMAN), IEEE TIP. It is a strong example of attention based multi branch hybrid design that improves generalization with efficiency; it helps contextualize your hybrid framing.

Ren et al., 2020; A Survey of Deep Active Learning ; comprehensive orientation to modern active learning

Schroder et al., 2020; Active Learning for Text Classification using Deep Neural Networks

Zhang et al., 2022; A Survey of Active Learning for Natural Language Processing

Reviewer #2: Thank you for the opportunity to review this work. The idea of using TF IDF features, a simple hybrid of four classifiers, and an active learning loop is practical, and testing across four datasets is useful. However, the paper needs more clarity before it can move forward. The contribution should be explained more clearly, as active learning with these models is already well explored. The experimental setup also needs complete details such as data splits, number of rounds, batch sizes, stopping rules, hyperparameters, and random seeds. Stronger comparisons and ablation studies are needed to show where the improvements truly come from. Since the goal is to reduce labeling cost, results showing accuracy versus labeled sample size would strengthen the claims. It would also help to report precision, recall, F1, and confusion matrices instead of only accuracy. The datasets should be described more transparently, with clear links and details on how the custom datasets were collected. Finally, the writing can be tightened and captions can better explain what readers should take away. With these improvements, the work can become much stronger.

**********

what does this mean?). If published, this will include your full peer review and any attached files.

If you choose “no”, your identity will remain anonymous but your review may still be made public.

**Do you want your identity to be public for this peer review?**  For information about this choice, including consent withdrawal, please see our Privacy Policy

Reviewer #1: No

Reviewer #2: **Yes:**  Ravi Shanker Sharma

**********

[NOTE: If reviewer comments were submitted as an attachment file, they will be attached to this email and accessible via the submission site. Please log into your account, locate the manuscript record, and check for the action link "View Attachments". If this link does not appear, there are no attachment files.]

To ensure your figures meet our technical requirements, please review our figure guidelines: https://journals.plos.org/plosone/s/figures

You may also use PLOS’s free figure tool, NAAS, to help you prepare publication quality figures: https://journals.plos.org/plosone/s/figures#loc-tools-for-figure-preparation.

NAAS will assess whether your figures meet our technical requirements by comparing each figure against our figure specifications.

# Author comment: Author response to Decision Letter 1

**DOI:** 10.1371/journal.pone.0355603.r002

28 Jan 2026

________________________________________

Response to Reviewers

Manuscript ID: PONE-D-25-44944

Title: A Novel Hybrid Model for Identifying the Most Informative Instances for Improving Text Data Classification

Dear Academic Editor and Reviewers,

We would like to sincerely thank the Academic Editor and the reviewers for their careful evaluation of our manuscript and for their constructive and insightful comments. We greatly appreciate the time and effort devoted to reviewing our work. The reviewers’ suggestions were highly valuable and significantly contributed to improving the clarity, rigor, and overall quality of the manuscript.

We are pleased to report that all comments and required revisions have been fully addressed. The manuscript has been carefully revised, expanded, and improved accordingly. Below, we provide a detailed point-by-point response to each reviewer’s comment, indicating how the manuscript has been revised.

________________________________________

Response to Reviewer #1

General Comment

We thank the reviewer for recognizing the practical relevance of the proposed hybrid active learning framework and its effectiveness for label-efficient text classification.

________________________________________

Comment 1: Baseline ambiguity between traditional ensemble and hybrid ensemble

Response:

We agree with the reviewer and have clarified this important distinction. The manuscript now explicitly explains that the Traditional Ensemble uses passive learning with randomly selected labeled samples, while the Hybrid Ensemble integrates the same base classifiers within a pool-based active learning loop using uncertainty sampling. This clarification has been added in Section 5.4, Page 10, second and fifth paragraphs after Tables (2-5) which reports the performance of the Traditional Ensemble model, the proposed Hybrid Ensemble model, and the BERT baselines.

“A clear and consistent trend emerges across all datasets. The proposed Hybrid Ensemble model consistently outperforms the Traditional Ensemble under all classical feature extraction methods. Among these methods, TF-IDF provides the highest accuracy and F1 scores for the hybrid model, confirming its superiority as the most effective representation in this study. In contrast, BOW features consistently result in the lowest performance, indicating their limited suitability for enhancing the predictive power of the hybrid ensemble.”

“Overall, the results confirm the robustness of the Hybrid Ensemble model. TF-IDF–based hybrid ensembles offer a lightweight yet highly effective alternative to transformer-based models, delivering competitive performance with substantially lower computational and annotation costs, making them particularly suitable for diverse text classification tasks.”

________________________________________

Comment 2: Specify active learning loop details

Response:

This comment has been fully addressed. We have explicitly described:

• Initial labeled set size (10%)

• Batch size per iteration (5%)

• Number of rounds (up to 15)

• Stopping criteria (performance plateau and labeling budget limit)

These details are now clearly presented in:

Sections 4.0 (Methodology): Second paragraph - complete description of pool-based active learning approach.

“The research utilizes a pool-based active learning approach. Unlabeled data is divided into batches that are selectively sampled for labeling by a human expert. After each batch is labeled, the algorithm is retrained on the labeled data. This iterative process of sampling, labeling, and retraining continues until model accuracy reaches an acceptable level. For sample selection, an uncertainty sampling method is used to choose the most informative instances to label from the unlabeled data pool. After each round of labeling, model accuracy is evaluated to determine if additional labeling is needed. Once sufficient accuracy is achieved, the final labeled dataset is used to train a classifier.”

Section 5.0. (Discussion): First three paragraphs describing the active learning process, stopping criteria, and implementation details.

“The active learning process began with an initial labeled set representing 10% of each dataset, selected randomly to establish a minimal supervised baseline. During each iteration, the model queried the most informative samples based on uncertainty sampling, using a batch size of 5% of the remaining unlabeled data. The active learning cycle continued for a total of 15 rounds, unless the stopping criteria were reached earlier.

A combined stopping strategy was adopted:

Performance Plateau Rule – the process stopped if the improvement in accuracy across three consecutive rounds was less than 0.5%.

Budget Limit Rule – active learning stopped when 50% of the dataset was labeled, ensuring realistic annotation constraints.”

Additionally, learning curves illustrating accuracy versus number of labeled samples have been generated and discussed to demonstrate label efficiency.

________________________________________

Comment 3: Report precision, recall, and F1 for imbalanced datasets

Response:

We have expanded the evaluation to include precision, recall, and F1-score for all datasets, with special emphasis on imbalanced data (SMS Spam dataset). Macro-averaged and weighted metrics are now reported and discussed in Section 4.5 and Section 5.0, providing a comprehensive evaluation.

Modification Locations in Manuscript:

• Abstract: Mentioning "accuracy, precision, recall, and F1 score" and "imbalanced classification scenarios"

“In particular, TF-IDF-based hybrid ensembles achieve the highest gains in accuracy, precision, recall, and F1 score, while requiring substantially fewer labeled instances. Furthermore, the proposed framework exhibits strong robustness in imbalanced classification scenarios, significantly improving minority class detection.”

• Section 4.5 (Text Classification): Completely new subsection titled "Evaluation for Imbalanced Datasets"

“Evaluation for Imbalanced Datasets

For imbalanced datasets, where one class significantly outnumbers others (as observed in the SMS Spam Collection dataset where spam messages constitute approximately 13% of all messages), we report precision, recall, and F1 scores for each class separately. Additionally, we calculate:

Macro-averaged scores - Treating all classes equally by computing the metric independently for each class and taking the average. This approach gives equal weight to each class regardless of support.

Weighted-averaged scores - Accounting for class imbalance by weighting each class's metric by its support (number of true instances). This provides a more representative overall score for imbalanced datasets.

The comprehensive reporting of these metrics ensures robust evaluation across different dataset characteristics and application requirements [47].”

• Section 4.5: in Tables 2-5: columns are added for Precision, Recall, and F1 Score for all models

• Section 5.0: New comprehensive section "Comprehensive Performance Analysis and Metrics Interpretation" with detailed analysis of Precision, Recall, and F1

“Comprehensive Performance Analysis and Metrics Interpretation

The evaluation of our proposed hybrid active learning model encompasses multiple performance metrics to provide a complete picture of classification performance. This comprehensive assessment is particularly crucial given the potential class imbalance present in some datasets, such as the SMS Spam Collection where spam messages constitute a minority class (approximately 13% of all instances).

Precision Analysis: Our results demonstrate that the hybrid model with active learning consistently achieves higher precision compared to traditional models across all datasets. The improvement in precision indicates that the model effectively reduces false positives. For instance, in the SMS dataset with TF-IDF features, precision improved from 0.76 (Traditional) to 0.85 (Hybrid AL), representing an 11.8% improvement. This is particularly important in spam detection scenarios where falsely classifying legitimate messages as spam (false positives) could result in users missing important communications.

Recall Analysis: The enhanced recall across all datasets shows better identification of true positive instances. In healthcare applications (HCR dataset), recall improved from 0.77 to 0.86 with the hybrid active learning model using TF-IDF, demonstrating the model's superior ability to correctly identify positive cases. High recall is critical in domains where missing positive instances (false negatives) carries significant consequences.

F1 Score Analysis: The F1 score, being the harmonic mean of precision and recall, confirms that the model maintains balanced performance between these two critical metrics. The consistent improvement in F1 scores across all datasets (ranging from 8% to 11% improvement) demonstrates that active learning not only enhances overall accuracy but also ensures balanced performance across different classes. This is particularly evident in the Financial Phrase Bank dataset where the F1 score improved from 0.78 to 0.85, indicating robust performance in sentiment classification tasks.”

• Figures 5-8: Updated bar charts showing all four metrics

• Conclusions: Emphasis on balanced performance and imbalanced dataset handling

“Beyond overall accuracy improvements, the proposed framework demonstrated strong robustness in handling imbalanced datasets. The active learning mechanism effectively prioritized informative instances from minority classes, leading to substantial gains in recall and balanced F1 scores without relying on additional resampling or cost-sensitive learning techniques.”

________________________________________

Comment 4: Explicitly mention human annotation

Response:

We have clarified that human annotation is simulated within the pool-based active learning framework, where a human oracle provides labels for the selected informative samples. This clarification has been added and described in Section 3.1 and Figure 3.

________________________________________

Comment 5: Benchmark against state-of-the-art methods including BERT

Response:

We thank the reviewer for this valuable suggestion. We have added BERT-based baselines (passive fine-tuning and active learning) and provided a detailed comparison with the proposed hybrid model. Experimental settings and hyperparameters are fully described, and results are reported and discussed in Section 4.5 and Tables 2–5.

Modification Locations in Manuscript:

• Abstract: Added "BERT-based embeddings" and "transformer-based approaches" mentions

• Tables 2-5: Two new rows added for each table - "BERT Traditional Ensemble" and "BERT Hybrid AL Model"

The provided tables (2–5) present the outcomes of applying various feature extraction techniques—Bag-of-Words (BOW), TF-IDF, Word2Vec—as well as the BERT-base model (bert-base-uncased) under both passive fine-tuning and active learning settings, across four datasets (HCR, Financial Phrase Bank, SMS Spam, and Textbook Dataset). Each table reports the performance of the Traditional Ensemble model, the proposed Hybrid Ensemble model, and the BERT baselines.

• After Tables (Section 4.5): Complete paragraph describing BERT experimental protocol, hyperparameters, and comparison methodology

“The incorporation of BERT provides a modern baseline for comparison. Passive BERT, fine-tuned on randomly sampled labeled subsets matching the labeling budgets (10%, 25%, 50%), generally surpasses the Traditional Ensemble model, reflecting the strength of pretrained transformer representations. However, under limited labeling budgets, its performance is comparable to or slightly below that of the Hybrid Ensemble with active learning. Integrating BERT into an active learning loop using uncertainty sampling improves its performance, but the Hybrid Ensemble remains competitive or superior in label efficiency. In several datasets, the hybrid model achieves similar or higher accuracy with significantly fewer labeled instances, highlighting its practical advantage in low-resource annotation scenarios.

BERT fine-tuning followed a standardized protocol for fair comparison: maximum sequence length = 128, learning rate = 2e-5, batch size = 16, optimizer = AdamW with weight decay 0.01, and up to 3 epochs with early stopping on validation loss. Both passive and active learning settings used identical train/validation/test splits and the same active learning configuration (initial seed size, batch selection per round, number of rounds, stopping criteria). Each reported result is averaged over 3–5 runs with different random seeds, and statistical comparisons were performed using paired tests across runs.”

• Conclusions: Statement comparing computational efficiency and cost-effectiveness versus transformer-based approaches

“From a computational perspective, the proposed framework offers a lightweight yet highly competitive alternative to transformer-based models. While pretrained language models such as BERT often require extensive labeled data and significant computational resources, the proposed hybrid active learning model achieves comparable or superior performance under constrained labeling budgets, with substantially lower computational overhead and annotation costs.”

________________________________________

Comment 6: Provide hyperparameters and implementation details

Response:

All implementation details, including feature extraction settings, classifier configurations, active learning parameters, and BERT fine-tuning hyperparameters, have been explicitly reported in Section 4.5 to ensure reproducibility.

Section 4.5 includes all implementation details Besides the following paragraph regarding BERT.

“BERT fine-tuning followed a standardized protocol for fair comparison: maximum sequence length = 128, learning rate = 2e-5, batch size = 16, optimizer = AdamW with weight decay 0.01, and up to 3 epochs with early stopping on validation loss. Both passive and active learning settings used identical train/validation/test splits and the same active learning configuration (initial seed size, batch selection per round, number of rounds, stopping criteria). Each reported result is averaged over 3–5 runs with different random seeds, and statistical comparisons were performed using paired tests across runs.”

________________________________________

Additional Literature

Response:

All recommended references have been carefully reviewed and incorporated into the Related Work section, including:

• Zafar et al. (2024)

• Ren et al. (2021)

• Schröder & Niekler (2020)

• Zhang et al. (2022)

These works are now properly cited and discussed in Section 2.0 (Related Work).

Modification Locations in Manuscript:

• Section 2.0 (Related Work): Two new paragraphs added at the end of the section discussing:

o Hybrid and attention-based architecture (Zafar et al., 2024)

o Modern active learning strategies and query design (Ren et al., Schröder & Niekler, Zhang et al.)

“Hybrid models, which combine multiple classifiers, have gained considerable attention due to their ability to leverage the strengths of different learning algorithms and improve generalization performance [20,21]. Recent research trends further emphasize the effectiveness of hybrid and attention-based architectures. For example, attention-driven multi-branch hybrid designs have been shown to enhance robustness and efficiency by adaptively fusing information from multiple components. Although primarily explored in computer vision, such principles provide useful insights for text classification and reinforce the motivation for adopting hybrid classifier ensembles.

In parallel, modern studies on active learning provide comprehensive guidance for designing effective query strategies. Active learning techniques focus on uncertainty sampling, diversity-based selection, and hybrid strategies that combine multiple criteria to identify the most informative samples [22,23]. These approaches have proven particularly effective for text classification, where labeling costs are high and data distributions are complex. Empirical evidence indicates that active learning can significantly reduce annotation effort while

Attachment Submitted filename: Response to Reviewers.docx

# Peer review: Decision Letter 1

**DOI:** 10.1371/journal.pone.0355603.r003

**Authors:** Agnieszka Konys

24 Jul 2026

A Novel Hybrid Model for Identifying the Most Informative Instances for Improving Text Data Classification

PONE-D-25-44944R1

Dear Dr. Abdelwahab,

We’re pleased to inform you that your manuscript has been judged scientifically suitable for publication and will be formally accepted for publication once it meets all outstanding technical requirements.

Within one week, you’ll receive an e-mail detailing the required amendments. When these have been addressed, you’ll receive a formal acceptance letter and your manuscript will be scheduled for publication.

An invoice will be generated when your article is formally accepted. Please note, if your institution has a publishing partnership with PLOS and your article meets the relevant criteria, all or part of your publication costs will be covered. Please make sure your user information is up-to-date by logging into Editorial Manager at Editorial Manager® and clicking the ‘Update My Information' link at the top of the page. For questions related to billing, please contact billing support.

If your institution or institutions have a press office, please notify them about your upcoming paper to help maximize its impact. If they’ll be preparing press materials, please inform our press team as soon as possible -- no later than 48 hours after receiving the formal acceptance. Your manuscript will remain under strict press embargo until 2 pm Eastern Time on the date of publication. For more information, please contact onepress@plos.org.

Kind regards,

Agnieszka Konys, Ph.D.

Academic Editor

PLOS One

Additional Editor Comments (optional):

Note from the Editorial Office. Please ignore any requests to cite papers from the reviewers that are issued as part of this decision.

Reviewers' comments:

Reviewer's Responses to Questions

**Comments to the Author**

Reviewer #2: All comments have been addressed

Reviewer #3: All comments have been addressed

**********

2. Is the manuscript technically sound, and do the data support the conclusions?

Reviewer #2: Yes

Reviewer #3: Yes

**********

3. Has the statistical analysis been performed appropriately and rigorously?

Reviewer #2: Yes

Reviewer #3: Yes

**********

4. Have the authors made all data underlying the findings in their manuscript fully available?

The PLOS Data policy

Reviewer #2: Yes

Reviewer #3: Yes

**********

5. Is the manuscript presented in an intelligible fashion and written in standard English?

Reviewer #2: Yes

Reviewer #3: Yes

**********

Reviewer #2: The manuscript presents a scientifically sound and well‑validated hybrid active learning framework for text classification. The authors have thoroughly addressed all reviewer comments by clarifying the methodological novelty, explicitly defining the active learning loop, expanding evaluation metrics (including precision, recall, and F1‑score for imbalanced datasets), and benchmarking against transformer‑based models such as BERT. Experimental design, dataset transparency, and implementation details are now clearly documented, ensuring reproducibility. The revised manuscript demonstrates strong methodological rigor, clear presentation, and well‑supported conclusions, meeting the scientific and editorial standards of PLOS ONE.

Reviewer #3: Reviewer Report

Recommendation: Accept after Minor Revision

The manuscript presents a hybrid active learning framework for text classification that integrates multiple machine learning classifiers with different text representation techniques. The study is well organized, the methodology is clearly explained, and the experimental evaluation on multiple benchmark datasets demonstrates the effectiveness of the proposed approach. The topic is timely and relevant to the fields of machine learning, active learning, and intelligent text analytics. The results support the conclusions drawn by the authors. I recommend acceptance after minor revision.

Minor Comments

Strengthen the literature review.

The Introduction and Related Work sections mainly cite studies on active learning for image analysis, object tracking, face recognition, and hyperspectral image classification (Refs. 38–47). Since the proposed framework is closely related to machine learning, entropy-based information measures, and data-driven predictive modeling, the authors should include more recent interdisciplinary references that discuss computational modeling and learning-based analytical frameworks. In particular, the following publications would improve the background and broaden the discussion:

Ghani, M.U. et al. QSPR Analysis of Distance-Based Structural Indices for Drug Compounds in Tuberculosis Treatment. Heliyon, 2024.

Ghani, M.U. et al. Computation of Entropy Measures for Metal–Organic Frameworks. Molecules, 2023.

Ghani, M.U. et al. Characterizations of Chemical Networks Entropies by K-Banhatti Topological Indices. Symmetry, 2023.

These references may be added to the related work section or used to replace less relevant references where appropriate.

Provide statistical significance analysis.

Although the proposed model achieves higher accuracy, precision, recall, and F1-score, the manuscript would be stronger if the authors reported statistical significance tests (e.g., paired t-test, Wilcoxon signed-rank test, or confidence intervals) to demonstrate that the observed improvements over competing methods are statistically meaningful.

Expand the discussion of computational efficiency.

The manuscript claims that the proposed framework is lightweight and cost-efficient compared with transformer-based approaches. Including quantitative comparisons of training time, inference time, memory consumption, or annotation cost would further support these claims and increase the practical value of the work.

Final Recommendation: The manuscript is technically sound and suitable for publication after addressing the above minor revisions.

**********

what does this mean?). If published, this will include your full peer review and any attached files.

If you choose “no”, your identity will remain anonymous but your review may still be made public.

**Do you want your identity to be public for this peer review?**  For information about this choice, including consent withdrawal, please see our Privacy Policy

Reviewer #2: **Yes:**  Ravi Shanker Sharma

Reviewer #3: No

**********

# Editor report: Acceptance letter

**DOI:** 10.1371/journal.pone.0355603.r004

**Authors:** Agnieszka Konys

PONE-D-25-44944R1

PLOS One

Dear Dr. Abdelwahab,

I'm pleased to inform you that your manuscript has been deemed suitable for publication in PLOS One. Congratulations! Your manuscript is now being handed over to our production team.

At this stage, our production department will prepare your paper for publication. This includes ensuring the following:

* All references, tables, and figures are properly cited

* All relevant supporting information is included in the manuscript submission,

* There are no issues that prevent the paper from being properly typeset

You will receive further instructions from the production team, including instructions on how to review your proof when it is ready. Please keep in mind that we are working through a large volume of accepted articles, so please give us a few days to review your paper and let you know the next and final steps.

Lastly, if your institution or institutions have a press office, please let them know about your upcoming paper now to help maximize its impact. If they'll be preparing press materials, please inform our press team within the next 48 hours. Your manuscript will remain under strict press embargo until 2 pm Eastern Time on the date of publication. For more information, please contact onepress@plos.org.

You will receive an invoice from PLOS for your publication fee after your manuscript has reached the completed accept phase. If you receive an email requesting payment before acceptance or for any other service, this may be a phishing scheme. Learn how to identify phishing emails and protect your accounts at https://explore.plos.org/phishing.

If we can help with anything else, please email us at customercare@plos.org.

Thank you for submitting your work to PLOS ONE and supporting open access.

Kind regards,

PLOS ONE Editorial Office Staff

on behalf of

Dr. Agnieszka Konys

Academic Editor

PLOS One
